# Polyorchidism: A Narrative Review on Clinical Manifestations, Diagnostic Challenges, Malignancy Risks, and Therapeutic Considerations

**DOI:** 10.7759/cureus.108046

**Published:** 2026-04-30

**Authors:** Abdulaziz Alzahrani, Bassam Bugis, Faisal Alslimah, Faisal alzahrani, Sultan AlKasim, Maher Moazin, Abdullah Alfakhri

**Affiliations:** 1 Urology, Prince Mansour Military Hospital, Al-Taif, SAU; 2 Urology, King Abdulaziz Hospital, Makkah, SAU; 3 Urology, King Fahad Medical City, Riyadh, SAU; 4 Urology, Faculty of Medicine, Al-Baha University, Al-Baha, SAU; 5 College of Medicine, King Saud University, Riyadh, SAU

**Keywords:** infertility, magnetic resonance imaging, polyorchidism, supernumerary testis, testicular cancer, testicular torsion, triorchidism, ultrasound

## Abstract

Polyorchidism, the presence of more than two testes, remains the rarest congenital anomaly of the male gonads, with fewer than 300 histologically or radiologically confirmed cases reported worldwide. Most supernumerary testes are discovered incidentally during imaging or surgery, yet their detection raises critical questions about malignancy risk, reproductive potential, and appropriate management. Current evidence indicates that approximately 90% of patients have three testes and two‑thirds show left‑sided duplication. About 75% of supernumerary testes are scrotal, roughly one‑fifth lie in the inguinal canal, and a small minority are abdominal or retroperitoneal. Inguinal hernia, cryptorchidism, and torsion are the most frequent associated anomalies, whereas hydrocele, varicocele, and renal agenesis occur less often. The pooled risk of neoplastic transformation ranges from 1% to 7% overall but climbs to 15% to 20% when the supernumerary testis is undescended. High‑resolution ultrasound is the diagnostic mainstay, while magnetic resonance imaging is indispensable for extrascrotal lesions and for clarifying drainage patterns. Contemporary practice increasingly favors surveillance of scrotal, anatomically normal, vas‑draining supernumerary testes, whereas extrascrotal, non‑draining, dysgenetic, or radiologically suspicious lesions warrant orchiectomy. Fertility outcomes remain poorly characterized. Isolated reports document both preserved spermatogenesis and severe oligoasthenoteratozoospermia, and endocrine profiles are usually normal. Advances in imaging have enabled conservative, fertility‑preserving management in many patients, but the absence of prospective registries, standardized follow‑up protocols, and molecular profiling limits reliable risk stratification. Multicenter collaborations are therefore essential to refine oncologic estimates, clarify reproductive implications, and establish evidence‑based guidelines for this exceptionally rare condition.

## Introduction and background

Polyorchidism was first reported by Blasius in 1670 and formally described by Lane in 1895 [1-3], yet it remains an enigmatic entity, with fewer than 300 confirmed cases in the medical literature [1,2]. Historically, the discovery of a supernumerary testis (SNT) led almost automatically to surgical removal because early authors believed the malignant potential was extremely high [2,4]. Over the past two decades, widespread access to high‑frequency ultrasound and improved magnetic resonance protocols has shifted clinical practice from excision toward individualized surveillance in selected patients [5,6]. However, clinical decision-making remains controversial because available evidence is largely derived from isolated case reports and small retrospective analyses, limiting reliable epidemiological and oncologic risk estimation [1,2].

Three fundamental questions continue to frame clinical debate: (1) whether anatomical or histological features can reliably stratify malignancy risk, (2) whether the additional testis contributes meaningfully to spermatogenesis or endocrine function, (3) and what constitutes optimal imaging and follow-up for lesions deemed suitable for preservation [1,2].

In this review, we examine embryological mechanisms, summarize the current understanding of clinical presentation and imaging, evaluate published evidence on neoplastic transformation, explore reported fertility outcomes, and propose a rational, risk‑adapted management framework. Remaining research gaps are emphasized so that future studies can be directed toward the most pressing uncertainties.

Methods

A comprehensive literature search was conducted using PubMed, Scopus, and Google Scholar databases. Relevant studies published in English were identified using combinations of keywords including “polyorchidism”, “supernumerary testis”, “testicular torsion”, ''testicular cancer", ''infertility”, ''magnetic resonance imaging”, ''ultrasound''.

Studies were selected based on their relevance to clinical presentation, diagnostic approaches, malignancy risk, fertility outcomes, and management strategies. Priority was given to studies providing detailed clinical, radiological, or histopathological data. Reference lists of included articles were also screened to identify additional relevant publications.

The selected studies were narratively synthesized, focusing on integrating findings across studies, identifying patterns, and highlighting areas of agreement, controversy, and gaps in the literature.

## Review

Embryology and pathogenesis

Normal testicular differentiation begins during the sixth gestational week when the primitive gonadal ridge transforms under the influence of the sex-determining region of the Y chromosome [7]. Concurrently, the mesonephric (Wolffian) ducts differentiate into the epididymis, vas deferens, and seminal vesicles under testosterone stimulation [7]. Fusion between the genital ridge and mesonephric duct epithelium creates the rete testis, a critical conduit for sperm transport [7,8].

Multiple embryological hypotheses have been proposed to account for SNT formation [8,9]. One theory suggests longitudinal or transverse cleavage of the primordial gonad before eighth‑week embryogenesis, resulting in two separate testicular anlagen that may share or possess independent mesonephric drainage [8]. A second hypothesis proposes atypical migration of primordial germ‑cell clusters that induces secondary testicular differentiation in adjacent tissue [8]. A third idea attributes polyorchidism to incomplete degeneration of mesonephric remnants that subsequently differentiate into additional testicular parenchyma [9]. Mechanical factors such as early intra‑uterine torsion or vascular accidents have also been theorized, but direct evidence remains limited [8]. Despite sporadic reports describing chromosomal abnormalities, including balanced translocations or mosaic aneuploidy, no reproducible genetic alteration has been consistently demonstrated to underlie polyorchidism [8,10].

Beyond these hypotheses, polyorchidism may represent a spectrum of gonadal ridge duplication disorders rather than a uniform anomaly [8]. The timing of embryologic division likely determines the degree of differentiation and drainage pattern, which, in turn, may influence fertility potential and oncologic risk [2,8,11]. Early division may permit independent epididymal and vasal development, whereas later cleavage may result in partially formed or non-draining remnants [8,11]. This temporal-developmental framework provides a plausible explanation for the anatomical heterogeneity described across classification systems [8].

**Figure 1:**
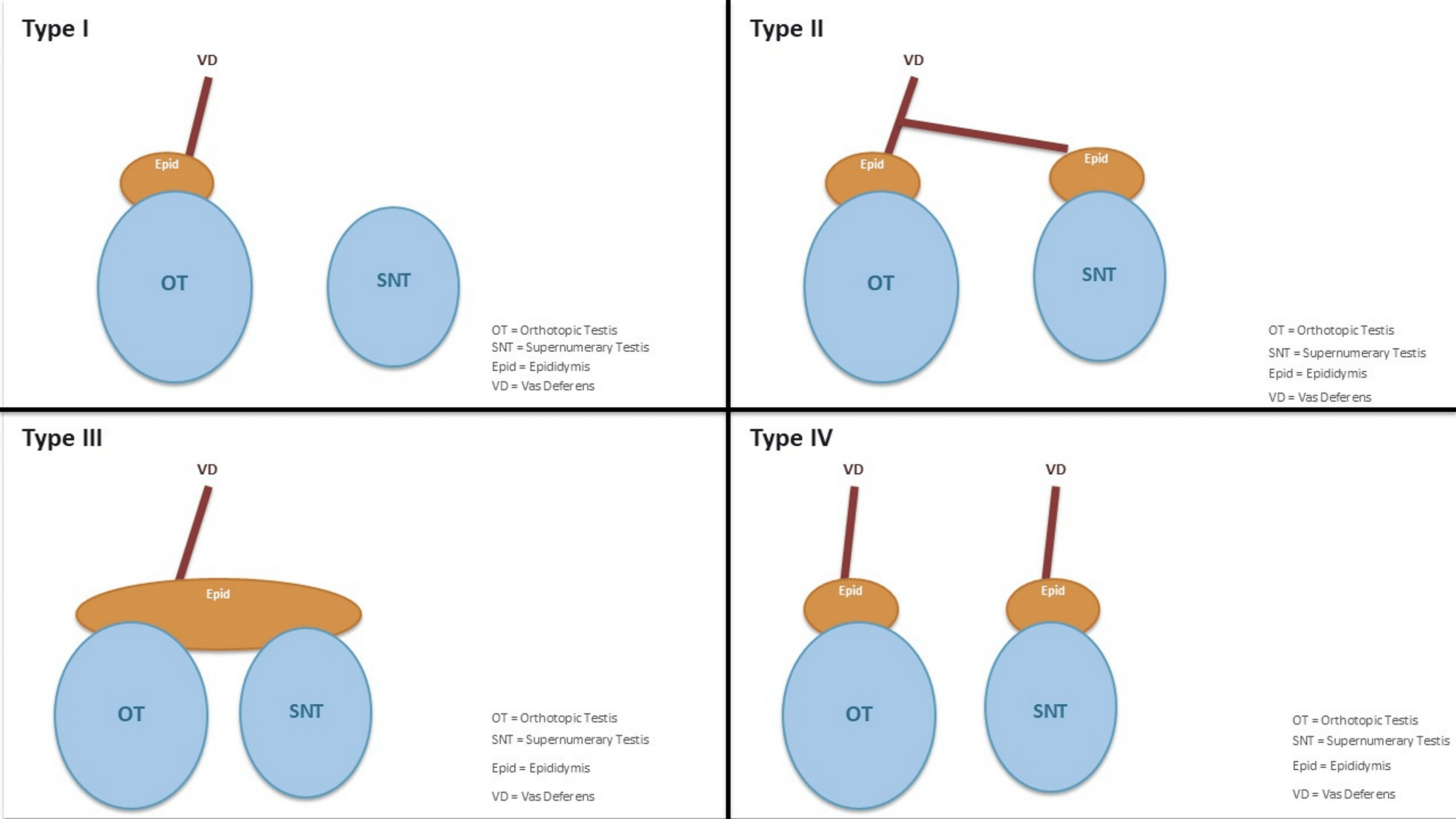
Leung classification of polyorchidism in 1988 The figure has been created by the author using Microsoft PowerPoint.

Leung published the first widely adopted anatomical classification in 1988 (Figure 1) [4]. He described four main types according to the presence or absence of epididymis and vas deferens: type I comprises an SNT without epididymis or vas, type II includes an SNT with its own epididymis but a shared vas, type III encompasses an SNT that shares both epididymis and vas with the orthotopic testis, and type IV represents complete duplication of the testis, epididymis, and vas deferens (Table 1) [4]. Singer subsequently introduced a functional classification in 1992 distinguishing draining (type A) from non-draining (type B) SNTs, emphasizing fertility and management implications [11]. Bergholz and Wenke refined this dichotomy in 2009 by dividing draining lesions into three patterns, depending on whether the vas and epididymis are duplicated or shared, and by distinguishing fibrotic, non‑draining remnants from those lacking any excretory ducts [2,12]. Functional classification carries practical implications because draining SNTs may contribute to fertility and appear to harbor a lower malignancy risk, whereas non‑draining structures tend to be dysgenetic and oncogenically vulnerable [2,13].

**Table 1:** Comparison of Leung classification of polyorchidism types in 1988 SNT, supernumerary testis

Feature	Type I	Type II	Type III	Type IV
Epididymis of SNT	Absent	Own (separate)	Shared with OT	Own (separate)
Vas deferens of SNT	Absent	Shared with OT	Shared with OT	Own (separate)
Drainage status	Non-draining	Draining	Draining	Draining
Degree of duplication	Testis only	Testis + epididymis	Testis only (attached)	Complete (testis + epididymis + Vas)
Fertility potential	None (no excretory pathway)	Present (via shared vas)	Present (via shared epididymis and vas)	Full (independent pathway)
Malignancy risk	Higher (non-draining, dysgenetic)	Lower (draining)	Lower (draining)	Lowest (fully functional)
Recommended management	Orchiectomy recommended	Surveillance if scrotal	Surveillance if scrotal	Surveillance if scrotal

Epidemiology and clinical manifestations

True prevalence in the general population remains unknown, and autopsy series and neonatal ultrasound screening programs estimate that SNT occurs in fewer than 5 per 100,000 male births [14]. A recent systematic review conducted in 2023 collated 190 reported cases and found that the median age at diagnosis was 23 years, although reported ages spanned from the neonatal period to the eighth decade of life [1]. Approximately two‑thirds of diagnoses occur before the age of thirty, reflecting detection during pediatric surgery for cryptorchidism or hernia and during young‑adult evaluation of scrotal pain or infertility [1,2].

Triorchidism, in which three testes are present, accounts for approximately 90% of reported cases [1,2]. Tetrorchidism forms the next most frequent configuration, representing roughly 6%. Instances of more than four testes are exceedingly rare; the largest documented example involves six separate testes [15]. Left‑sided duplication predominates, with about 65% of SNTs located on the left, a pattern attributed to asymmetric descent dynamics and earlier obliteration of the processus vaginalis on the right [9]. Bilateral polyorchidism, characterized by an extra testis on each side, has been described in fewer than four published cases [1].

Concomitant urogenital anomalies are common. Inguinal hernia appears in roughly a quarter to one‑third of patients, whereas cryptorchidism affects between 22% and 40% [2]. Testicular torsion complicates 7% to 15% of lesions. Hydroceles, varicoceles, hypospadias, epididymal cysts, and ipsilateral renal agenesis are less frequent but well-documented [1,16].

Clinical presentation varies. Approximately 60% of SNTs are discovered incidentally during imaging for unrelated complaints or during open surgery [1]. When symptoms exist, they tend to correspond to associated complications. A firm, painless nodule in the scrotum often prompts radiological evaluation for suspected neoplasm and subsequently reveals an SNT. Sudden scrotal pain may herald torsion [2,17], particularly if the supernumerary organ possesses a pedunculated mesentery. Inguinal SNTs manifest as groin swellings that mimic hernias or lymphadenopathy [2]. Infertility work‑ups occasionally expose unsuspected SNTs when semen analysis is abnormal, and ultrasound is ordered [1,18]. Finally, traumatic disruption of a multilobulated testicular contour can lead to incidental discovery.

Diagnostic imaging and related pitfalls

High‑frequency sonography is the cornerstone of initial assessment [17]. An SNT generally appears as an ovoid, homogeneous structure with echogenicity identical to that of the orthotopic testis [5]. Mean longitudinal diameter ranges between 15 and 20 mm, although organs of equal size to the native testis have been reported [5,6,17]. A thin, echogenic tunica albuginea, if present, demarcates the parenchyma [5]. Color Doppler interrogation usually demonstrates symmetric arterial and venous flow [5]. The absence of a mediastinum testis or significant reduction in vascularity raises suspicion for dysgenesis, torsion, or fibrosis [5].

Despite high sensitivity, ultrasound may be confounded by several entities. A bilobed testis presents as a clefted but continuous gonad and lacks a discrete tunical covering [17]. Splenogonadal fusion can mimic an ectopic testis but shows reticuloendothelial uptake on nuclear scintigraphy and demonstrates splenic vasculature [19]. Adenomatoid tumors, paratesticular sarcomas, hernia sacs, and epididymal cysts may also resemble SNTs under certain circumstances [19].

When an SNT resides outside the scrotum or when ultrasound characteristics are equivocal, magnetic resonance imaging (MRI) is invaluable [19]. On T1‑weighted sequences, testicular tissue is isointense to skeletal muscle, whereas on T2‑weighted images, it is hyperintense relative to muscle but hypointense relative to pure fluid [6]. Homogeneous post‑gadolinium enhancement confirms viability. Diffusion‑weighted imaging shows high signal and low apparent‑diffusion‑coefficient values consistent with tightly packed seminiferous tubules; lower ADC values or heterogeneous enhancement should prompt oncologic concern [6]. MRI images also depict epididymal and vasal anatomy with clarity, allowing the clinician to classify the SNT according to drainage pattern [6].

Although high-resolution ultrasound and MRI are considered highly reliable in identifying SNTs, formal sensitivity and specificity data are lacking due to the rarity of the condition and the absence of large-scale diagnostic studies. Diagnosis is therefore primarily based on the demonstration of imaging characteristics identical to normal testicular tissue rather than validated quantitative performance metrics.

Computed tomography rarely contributes to diagnosis, except when intra‑abdominal SNTs are suspected in conjunction with other congenital abnormalities. Technetium‑99m pertechnetate scintigraphy is seldom used today [19]. Positron‑emission tomography with fluorodeoxyglucose is reserved for staging confirmed testicular tumors [19].

Malignancy risk and histopathological spectrum

The potential for malignancy is the greatest concern associated with SNTs. In Bergholz and Wenke’s meta‑analysis comprising 140 pooled cases, nine tumors were identified, yielding a crude incidence of 6.4%, although reporting bias cannot be excluded [2]. Subsequent syntheses have reproduced a pooled estimate ranging between 1% and 7%. Importantly, location substantially modulates risk [1,2]. Scrotal, draining SNTs have yielded malignancies rarely, with reported proportions under 2%, whereas inguinal or abdominal SNTs display malignant transformation in approximately one in five cases [20]. This distribution parallels the established observation that undescended orthotopic testes carry a three‑ to eight‑fold higher risk of germ cell tumors than their scrotal counterparts [21].

Seminoma represents nearly two‑thirds of SNT tumors and typically presents in the third to fourth decade of life [1,2]. Non‑seminomatous germ cell tumors such as embryonal carcinoma, choriocarcinoma, and teratoma comprise the remaining cases [1,2]. Pediatric rhabdomyosarcoma has been described only once. Comprehensive genomic analysis of neoplastic SNTs is absent; therefore, it remains unknown whether these tumors harbor the same molecular alterations seen in conventional germ‑cell neoplasia, specifically the characteristic gain of chromosome 12p or activating mutations in the KIT gene [22].

Given the paucity of prospective data, follow‑up strategies have been extrapolated from cryptorchidism literature [1,2]. Most authors advise surveillance for scrotal, draining SNTs consisting of physical examination and ultrasound every 6 to 12 months; the acquisition of serum tumor markers - alpha‑fetoprotein, beta‑human‑chorionic‑gonadotropin, and lactate dehydrogenase - annually is optional but prudent when radiological findings change [23]. For non‑scrotal SNTs left in situ because of parental preference or surgical risk, imaging intervals should not exceed six months. Some clinicians advocate lifelong observation, citing tumors reported more than two decades after initial recognition [20].

Fertility and endocrine function

Whether an SNT enhances, impairs, or exerts no influence on reproductive capacity remains an unresolved issue. Histological evaluation of excised SNTs reveals normal seminiferous architecture in roughly 40%, maturation arrest in a quarter, and pronounced fibrosis in the remaining specimens [24]. Cases in which patients fathered children before or after detection of a scrotal, vas‑connected SNT suggest that preserved fertility is possible [23]. Conversely, Fang and colleagues recently reported severe asthenoteratozoospermia in a man with a scrotal triorchidism configuration despite otherwise normal endocrine parameters and no varicocele, indicating that the mere presence of additional testicular tissue does not guarantee adequate spermatogenesis [10]. Azoospermia has also been documented in polyorchidism linked to diffuse testicular dysgenesis [18].

Most hormonal studies describe normal concentrations of testosterone, luteinizing hormone, and follicle‑stimulating hormone, reinforcing the notion that endocrine sufficiency is generally maintained [25]. Nonetheless, long‑term surveillance data for pubertal progression and adult endocrine stability are lacking [1,2].

Assisted‑reproduction techniques provide an additional avenue when fertility is compromised. Micro‑dissection testicular sperm extraction has retrieved viable sperm from dysgenetic SNTs, enabling intracytoplasmic sperm injection in previously published cases [26]. When orchiectomy is indicated, cryopreservation of spermatozoa harvested intra‑operatively should be discussed, particularly for adolescents and men with contralateral atrophy [23].

Therapeutic considerations

During most of the twentieth century, surgeons excised every extra testis, largely because they were encountered during open surgery and malignancy seemed inevitable [24]. The modern approach is nuanced and risk‑adapted. When an SNT is scrotal, appears sonographically identical to the native testis, drains via a demonstrable vas deferens, and shows no laboratory or radiological evidence of neoplasia, many experts recommend conservative follow‑up [11,27]. The rationale rests on three pillars: reported malignant transformation in this subset is exceedingly rare, fertility potential might be augmented, and surgical removal carries a small but real risk of compromising vascular supply to the native testis [1,2,11,27].

In contrast, inguinal or abdominal SNTs almost universally warrant excision because their malignant propensity is dramatically higher and because reliable self‑examination or ultrasonographic evaluation is more difficult [1,2,20,27]. When a scrotal SNT exhibits heterogeneous echotexture, internal calcification, increasing size, or elevated tumor markers, radical inguinal orchiectomy is indicated, following standard protocols for suspected testicular cancer [1,2,12,27].

Surgical technique mirrors conventional orchiectomy. An inguinal incision affords control of the spermatic cord. Careful microvascular dissection preserves arterial inflow and venous drainage to the orthotopic testis [1,11,27]. Frozen‑section analysis may be considered when oncologic suspicion exists, but parenchymal preservation is desirable, although the accuracy of intra‑operative histology in germ cell tumors is not absolute [14,22,27]. Post‑operative complications are infrequent and generally limited to hematoma or minor wound infection. Long‑term androgen deficiency has not been reported in patients retaining at least one functional testis [1,2,27,28].

Discussion

The literature surrounding polyorchidism is constrained by inherent issues related to its infrequency [1,2,15]. Consequently, there is a lack of comparative data, and case reports dominate the literature. Additionally, it is more probable that cases presenting with symptoms such as torsion or malignancy will be reported, which would create reporting biases most likely contributing to an overestimation of risk [1,2]. However, despite these limitations, multiple systematic reviews provide evidence supporting two major tenets [1,2,8]. Firstly, the anatomical location of the SNT has a strong influence on its malignant potential [1,2,20,21]. Lesions located within the scrotum which drain into the reproductive system generally exhibit low-grade behavior. Conversely, those SNTs located outside of the scrotum or that do not drain into the reproductive system have a high degree of histologic similarity to their cryptorchid counterparts. Both have similar degrees of dysgenesis and oncogenetic predisposition [1,2,11,20,21]. Secondly, the ability to characterize SNTs using current radiographic modalities allows physicians to determine which patients may safely avoid surgical intervention [1,5,6,11,17,24].

Recent literature increasingly supports conservative management for uncomplicated, scrotal polyorchidism, particularly in pediatric and adolescent populations. For instance, Aldughiman et al. demonstrated successful conservative management in a prepubertal boy with a scrotal SNT, utilizing regular ultrasound follow-up and self-examination without adverse outcomes [29], which mirrors recommendations that asymptomatic draining scrotal SNTs may be safely monitored [30,31]. However, surgical exploration remains a valuable tool when imaging is inconclusive or when parental preference strongly favors histological confirmation and definitive management [31]. Therefore, the decision as to whether to use conservative management or surgical intervention must be based on each case individually because while there are some oncologic risks associated with these conditions, there is also the possibility of saving functioning testicular tissue [32,33].

There are significant research gaps remaining in polyorchidism; there is still insufficient quality data that can be used to help make decisions regarding the care of patients with this disease, and at present, there is no evidenced based agreement on how best to manage polyorchidism [1,15]. The current literature includes many isolated case reports and small retrospective series. These types of studies prevent the accurate estimation of the true incidence of polyorchidism and allow for the distortion of oncologic risk estimates due to possible reporting biases [1,2]. Due to the limited number of prospective semen studies available, fertility counseling continues to be challenging in relation to assessing reproductive potential because each individual case has demonstrated variable results, ranging from normal spermatogenesis to severely impaired spermatogenesis [10,24]. The lack of a standardized approach to managing polyorchidism is evident through the continued necessity for authors to provide their own proposed treatment algorithm [2,32]. The psychological and social effects of this condition, including concerns about body image and the anxiety that patients experience related to cancer, have yet to be formally explored and represent a significant gap in the care provided to those affected [1,2].

Furthermore, the psychological impact of polyorchidism and its management should not be overlooked. Patients may experience anxiety regarding both the risk of malignancy and their future fertility. Shared decision-making, supported by clear radiological evidence and patient education, is crucial in these cases [7,11].

Future directions

Building a prospective, multicenter registry would allow accurate determination of incidence, natural history, and malignancy risk across anatomical subtypes. Integrating detailed imaging phenotypes, operative findings, histology, and long‑term oncological and fertility outcomes would generate data capable of informing robust guidelines. Molecular profiling of tumorous SNTs could clarify whether typical germ cell tumor pathways are involved or whether distinct mechanisms render these rare neoplasms amenable to targeted therapy. Development of standardized imaging protocols, including modality choice, surveillance interval, and criteria for discharge, would harmonize care. Finally, assessing patient‑reported outcomes such as sexual function, body image, and psychological well‑being would ensure that management strategies align with the priorities of affected individuals.

## Conclusions

Polyorchidism is an exceptionally rare but increasingly recognized congenital anomaly, largely due to widespread ultrasound utilization. In most cases, the condition follows a benign course, particularly when the supernumerary organ is scrotal and drains via the vas deferens. Conservative monitoring with periodic imaging and patient education constitutes a safe strategy for this cohort. Conversely, extrascrotal SNTs should be excised because their malignant potential mirrors that of cryptorchid gonads. Current literature suggests that fertility is usually preserved, although high‑quality evidence is lacking. Prospective multinational registries, systematic semen and hormonal studies, and genomic exploration of neoplastic cases are urgently needed to refine risk stratification and inform consensus guidelines.
